# Magnetic CuFe_2_O_4_ Nanoparticles with Pseudocapacitive Properties for Electrical Energy Storage

**DOI:** 10.3390/molecules27165313

**Published:** 2022-08-20

**Authors:** Wenyu Liang, Wenjuan Yang, Sadman Sakib, Igor Zhitomirsky

**Affiliations:** Department of Materials Science and Engineering, McMaster University, Hamilton, ON L8S4L7, Canada

**Keywords:** spinel, dispersant, copper, iron, oxide, supercapacitor, magnetic

## Abstract

This investigation is motivated by increasing interest in the development of magnetically ordered pseudocapacitors (MOPC), which exhibit interesting magnetocapacitive effects. Here, advanced pseudocapacitive properties of magnetic CuFe_2_O_4_ nanoparticles in negative potential range are reported, suggesting that CuFe_2_O_4_ is a promising MOPC and advanced negative electrode material for supercapacitors. A high capacitance of 2.76 F cm^−2^ is achieved at a low electrode resistance in a relatively large potential window of 0.8 V. The cyclic voltammograms and galvanostatic charge–discharge data show nearly ideal pseudocapacitive behavior. Good electrochemical performance is achieved at a high active mass loading due to the use of chelating molecules of ammonium salt of purpuric acid (ASPA) as a co-dispersant for CuFe_2_O_4_ nanoparticles and conductive multiwalled carbon nanotube (MCNT) additives. The adsorption of ASPA on different materials is linked to structural features of ASPA, which allows for different interaction and adsorption mechanisms. The combination of advanced magnetic and pseudocapacitive properties in a negative potential range in a single MOPC material provides a platform for various effects related to the influence of pseudocapacitive/magnetic properties on magnetic/pseudocapacitive behavior.

## 1. Introduction

The ability to combine advanced electrical and magnetic properties in a single material holds great potential for the development of novel devices based on the control of electrical/magnetic properties in magnetic/electric fields. Various materials combining ferroelectric and magnetic properties [1], also called multiferroics [2], have been developed. Materials of different types such as perovskites, boracites, hexagonal manganates and materials of the BaMeF_4_ (Me = Mn, Fe, Co, Ni) and hexagonal BaTiO_3_ families have been investigated [1]. Interesting physical phenomena were observed in such materials, such as linear and nonlinear magnetoelectric effects, anomalies of dielectric/magnetic properties near magnetic/ferroelectric phase transition temperatures, polarization reversal in a magnetic field and magnetization reversal in an electric field [1]. However, it is challenging to achieve a combination of ferroelectric and ferri- or ferromagnetic properties in a single crystalline phase at room temperature [1]. High electrical resistivity is important to achieving ferroelectric polarization in an electric field. Many multiferroic materials exhibit relatively low resistivity, and investigation into their ferroelectric polarization presents difficulties. A high dielectric constant is usually observed in soft ferroelectrics at low voltages; an increase in applied voltage results in a significant reduction in the dielectric constant. Various multiferroic materials, such as BiFeO_3_, exhibit antiferromagnetic properties at room temperature, while other materials show weak ferri- or ferromagnetism at low temperatures [1]. Oxide materials offer benefits of higher resistivity; however, room-temperature ferroelectricity has not been observed in advanced magnetic oxides, such as spinels, garnets or hexagonal ferrites.

Recently, significant interest has been generated in magnetically ordered pseudocapacitors (MOPC) [3], which combine advanced magnetic and electrical charge storage properties. Pseudocapacitive properties of such materials are related to the redox reactions of metal ions. The capacitance of pseudocapacitive materials is many orders of magnitude larger than that of ferroelectric materials [4]. Many pseudocapacitive materials and composites exhibit nearly rectangular cyclic voltammograms and linear chronopotentiometry dependences, indicating their ideal capacitive behavior [5,6,7]. In contrast to ferroelectric materials, low resistance is beneficial for charging supercapacitor materials [8]. The reduction in particle size of ferroelectric materials to the nanometric scale usually results in a drastic reduction in spontaneous polarization and dielectric constant. In contrast, a significant increase in pseudocapacitive properties is achieved in nanostructured MOPC materials [5].

Ferrimagnetic spinels [9,10] and hexagonal ferrites [11] show promising electrical charge storage properties based on redox reactions. Interesting phenomena have been observed in MOPC materials, such as the enhancement of charge storage properties in magnetic fields [12,13,14]. Spinel materials have shown good capacitive properties in various aqueous electrolytes [15]. High areal capacitance has been reported for Fe_3_O_4_ spinel electrodes [16] in a Na_2_SO_4_ electrolyte. CoFe_2_O_4_ has been found to be another MOPC material showing high capacitance in KOH and NaOH electrolytes [17,18,19]. NiFe_2_O_4_ has shown good capacitive performance in KOH electrolytes [20].

CuFe_2_O_4_ is a promising MOPC material which exhibits ferrimagnetic properties. The saturation magnetization of this material is influenced by cation distribution in tetrahedral (T) and octahedral (O) cites [21,22] of the spinel structure [(1 − x)Cu^2+^(x)Fe^3+^]^T^[(x)Cu^2+^(2 − x)Fe^3+^]O_4_. It is expected that the reduction of Cu^2+^ and Fe^3+^ ions in the negative potential range can result in pseudocapacitive charge storage properties. It is in this regard that Cu^2+^ ions can be reduced [23,24,25] to Cu^+^, whereas Fe^3+^ ions can be reduced [26,27,28] to Fe^2+^. Electrochemical reduction of Cu^2+^ and Fe^3+^ ions can result in changes in their magnetic moments and influence superexchange interactions of the ions distributed in (T) and (O) positions of the crystal structure. This can potentially result in a decrease or increase in total magnetization, which depends on the magnetization of the individual sublattices. Therefore, the investigation of capacitive properties of CuFe_2_O_4_ in a negative potential range can potentially result in interesting phenomena related to the influence of pseudocapacitive/magnetic properties on magnetic/pseudocapacitive behavior.

Significant advances [29,30,31,32] in supercapacitor technology have been achieved by the development of advanced fabrication methods which allow good utilization of fundamental material properties using advanced design. Colloidal methods offer many benefits for the fabrication of supercapacitor electrodes. The success in the application of colloidal methods is inevitably related to the development of advanced dispersant molecules and advanced dispersion mechanisms [33,34]. Colloidal strategies have a high potential for the development of supercapacitor electrodes. However, it is challenging to disperse ferrimagnetic nanoparticles and prevent their agglomeration due to van der Waals and magnetic attraction forces.

The goal of this investigation is to fabricate and test pseudocapacitive properties of CuFe_2_O_4_ in a negative potential range. We investigate the magnetic and capacitive properties of CuFe_2_O_4_ nanoparticles and demonstrate that high areal capacitance can be achieved in a negative potential range in a neutral Na_2_SO_4_ electrolyte. The electrodes show nearly ideal pseudocapacitive behavior. High areal capacitance in a relatively large potential window is achieved at a low impedance, which is critical for practical applications. The ability to achieve high areal capacitance in the Na_2_SO_4_ electrolyte in a negative potential range is promising for the fabrication of advanced anodes for supercapacitor devices.

The experimental results presented in this paper indicate that good capacitive behavior can be achieved by the development of an advanced colloidal strategy for electrode fabrication. The problem of the strong agglomeration of ferrimagnetic CuFe_2_O_4_ nanoparticles is addressed using an advanced dispersant which allows for strong tridentate chelating bonding to the metal atoms on the particle surface. We demonstrate that the colloidal strategy developed in this investigation is a key factor for achieving the superior capacitive behavior of CuFe_2_O_4_ nanoparticles with a record-high areal capacitance for this material. Moreover, the CuFe_2_O_4_ electrodes prepared using this strategy are on a par with the most promising negative electrodes for asymmetric supercapacitors.

## 2. Results

**Figure 1:**
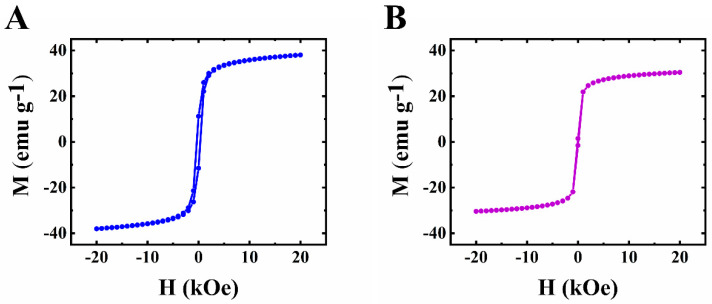
Magnetization (M) versus magnetic field (H) for CuFe_2_O_4_ at (**A**) 5 K and (**B**) 293 K.

CuFe_2_O_4_ is a ferrimagnetic material. Magnetization versus magnetic field dependence at a temperature of 5K (Figure 1A) showed magnetic hysteresis. However, the hysteresis was very small at 293K (Figure 1B). Magnetic measurements indicated the ferrimagnetic behavior of CuFe_2_O_4_. Saturation magnetization and the coercive field of CuFe_2_O_4_ decreased with increasing temperature in agreement with other investigations [35,36].

**Figure 2:**
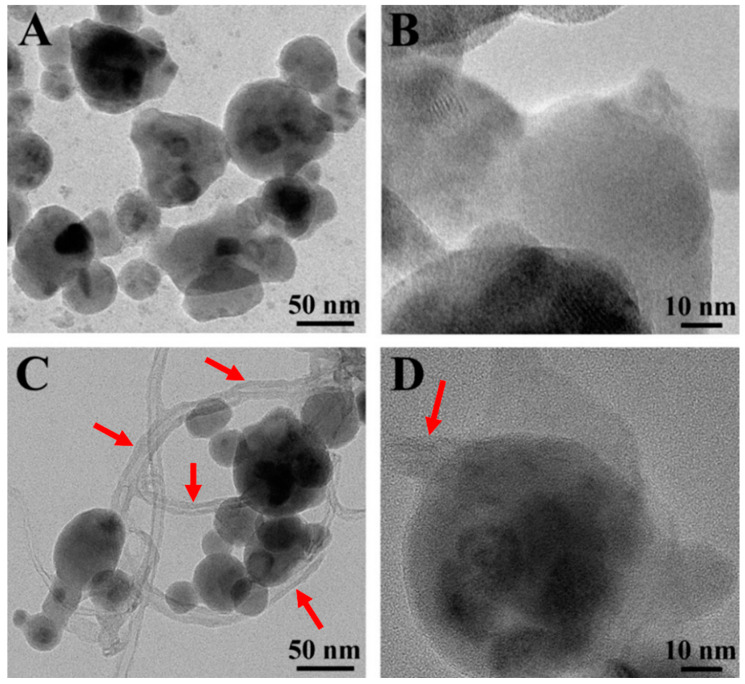
TEM images for (**A**,**B**) CuFe_2_O_4_ and (**C**,**D**) CFO-20. Arrows show MCNTs.

Figure 2 shows TEM images of the particles and a composite used in this investigation. The size of the particles was below 100 nm. Such particles were used for the fabrication of supercapacitor electrodes by a colloidal technique using MCNTs as conductive additives. The masses of MCNTs in the CuFe_2_O_4_-MCNT composites CFO-0, CFO-10, CFO-20 and CFO-30 were 0, 10, 20 and 30 wt.%, respectively. It will be shown below that dispersion and the efficient mixing of CuFe_2_O_4_ and MCNT had a tremendous impact on the pseudocapacitive properties of the CuFe_2_O_4_-based electrodes.

It is known that nanoparticles are prone to agglomeration due to their high surface energy and van der Waals attraction forces. Moreover, the magnetic interactions of the CuFe_2_O_4_ particles also promote their aggregation. Therefore, it is challenging to achieve a good dispersion of CuFe_2_O_4_ nanoparticles. Another challenge is the good dispersion of MCNTs. The co-dispersion of CuFe_2_O_4_ particles and MCNTs is critical for their efficient mixing and fabricating composite electrodes with high conductivity. In such composites, well-dispersed MCNTs must provide a conductive path to the CuFe_2_O_4_ particles and facilitate electrochemical redox reactions. As-received MCNTs formed agglomerates with a typical size of ~500 μm. The SEM images of such agglomerated MCNTs were presented in a previous investigation [37].

**Figure 3:**
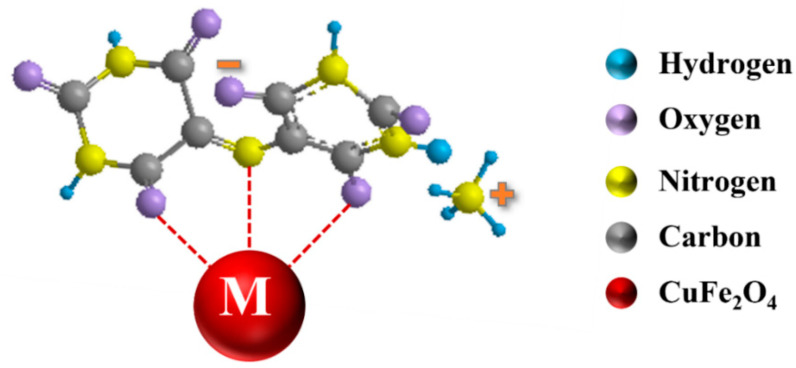
Adsorption of ASPA on CuFe_2_O_4_ particles, involving chelation of surface atoms (M = Cu or Fe).

**Figure 4:**
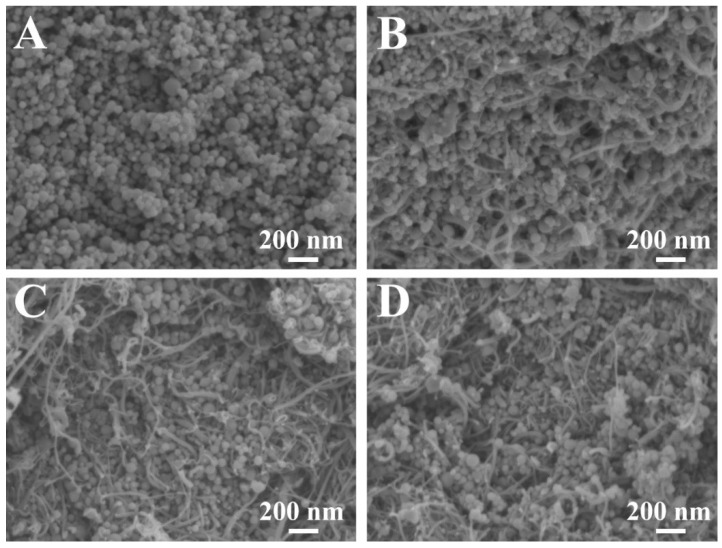
SEM images of (**A**) CFO-0, (**B**) CFO-10, (**C**) CFO-20 and (**D**) CFO-30 electrodes.

**Figure 5:**
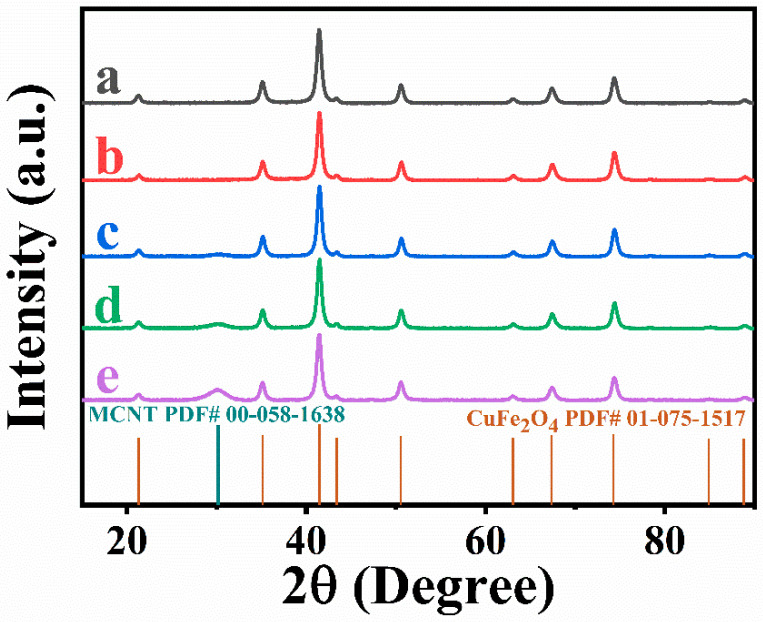
XRD data for (a) as-received CuFe_2_O_4_ and electrodes: (b) CFO-0, (c) CFO-10, (d) CFO-20 and (e) CFO-30.

The choice of dispersants plays a crucial role in the nanotechnology of composites. It is important to find a dispersant suitable for the dispersion of both CuFe_2_O_4_ particles and MCNTs. The dispersant must be strongly adsorbed on CuFe_2_O_4_ particles and MCNT, because non-adsorbed dispersant can promote agglomeration. Therefore, CuFe_2_O_4_ particles and MCNTs must be co-dispersed using a co-dispersant. The anchoring groups of dispersants play a vital role in their adsorption on inorganic particles. Recent studies [38] have shown that monodentate bonding provides relatively weak adsorption and new types of dispersants have been developed with bidentate chelating or bridging bonding. Such dispersants showed superior adsorption and facilitated the development of advanced nanocomposites and film deposition technologies [38]. These studies highlighted the advantages of charged dispersants containing chelating anchoring groups which facilitated dispersant adsorption by creating complexes with metal atoms on the particle surface [38,39,40]. Moreover, it was found that redox-active dispersants containing chelating groups could facilitate charge transfer between oxide particles and conductive additives or current collectors and increase pseudocapacitance [41]. In this investigation, we examined properties of ASPA molecules for the co-dispersion of CuFe_2_O_4_ particles and MCNTs. It is known that ASPA exhibits redox-active properties [42] in a negative potential range and forms complexes with Cu, Fe, Ni, Zn, Co and other metals [43]. The complex formation involved a tridentate bonding [44]. It was suggested that similar complexes (Figure 3) can be formed with Cu and Fe atoms on the surface of CuFe_2_O_4_ particles. It was found that the adsorbed negatively charged ASPA molecules allowed for electrostatic particle repulsion and the improved suspension stability of CuFe_2_O_4_ particles. Moreover, a good suspension stability of MCNTs was achieved in the presence of ASPA. It is suggested that ASPA adsorption on MCNTs involved hydrophobic interactions of the side walls of the MCNTs with barbiturate-like rings [44] of the ASPA molecules. The electrostatic co-dispersion of the CuFe_2_O_4_ particles and MCNTs facilitated their improved mixing. Small ASPA molecules efficiently separated individual MWCNs by breaking original large agglomerates and the improved contact of CuFe_2_O_4_ particles and MCNTs was achieved (Figure 2C,D). Previous investigations have shown that small organic dispersants facilitate carbon nanotube dispersion by an unzipping mechanism [45]. SEM images of CuFe_2_O_4_ electrodes showed non-agglomerated nanoparticles (Figure 4A). The composite electrodes showed MCNTs dispersed between CuFe_2_O_4_ particles (Figure 4B–D). EDS mapping confirmed the uniform distribution of CuFe_2_O_4_ and MCNTs in the composite (Supplementary Information, Figure S1 in Supplementary Material). A good dispersion of MCNTs was a key factor for the enhanced contact of CuFe_2_O_4_ particles with the conductive MCNT network and enhanced electrode performance. Figure 5 shows XRD patterns of as-received CuFe_2_O_4_ and CFO-0, CFO-10, CFO-20 and CFO-30 composite electrodes. The XRD data confirmed that composites contained CuFe_2_O_4_ and MCNTs.

**Figure 6:**
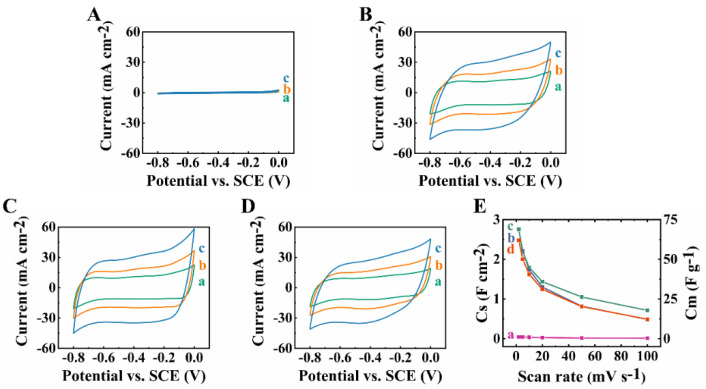
(**A**–**D**) CVs at scan rates of (a) 5, (b) 10 and (c) 20 mV s^−1^ for (**A**) CFO-0, (**B**) CFO-10, (**C**) CFO-20 and (**D**) CFO-30 electrodes; (**E**) capacitance versus scan rate for (a) CFO-0, (b) CFO-10, (c) CFO-20 and (d) CFO-30 electrodes.

**Figure 7:**
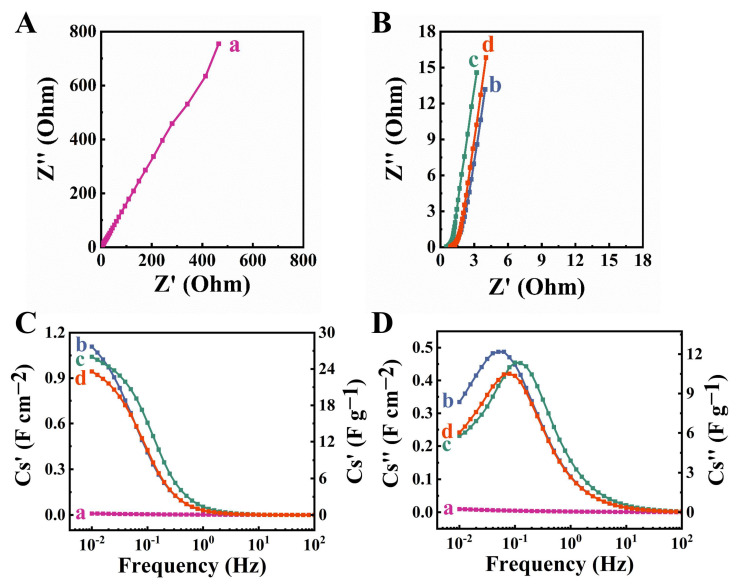
(**A**,**B**) Nyquist plots of impedance; (**C**) real and (**D**) imaginary part of complex capacitance derived from the impedance data versus frequency for (a) CFO-0, (b) CFO-10, (c) CFO-20 and (d) CFO-30 electrodes.

CV studies of the CFO-0 electrodes showed very low currents, indicating poor pseudocapacitive properties (Figure 6A). The low currents and small CV areas were observed due to the low electronic conductivity of CFO-0. High electronic conductivity is one of the key factors for the efficient charge–discharge of pseudocapacitive materials. The addition of conductive MCNT and the efficient co-dispersion of CuFe_2_O_4_ and MCNT resulted in the capacitive behavior of CFO-10, CFO-20 and CFO-30 electrodes (Figure 6B–D). CFO-10, CFO-20 and CFO-30 electrodes showed nearly rectangular-shape CVs, indicating good pseudocapacitive behavior and fast kinetic charge–discharge reactions. The observed increase in current with increasing scan rate was another indicator of good pseudocapacitive properties of the electrodes. It is suggested that capacitive properties of CFO in the negative potential range can result from Cu^2+^ reduction [23,24,25] to Cu^+^, and Fe^3+^ reduction [26,27,28] to Fe^2+^. Figure 6E shows capacitance versus scan rate dependencies. CFO-0, CFO-10, CFO-20 and CFO-30 electrodes showed capacitances of 0.04, 2.75, 2.76 and 2.48 F cm^−2^ at a scan rate of 2 mV s^−1^ and capacitance retention of 25, 17.8, 25.7 and 19.8% at a scan rate of 100 mV s^−1^. CV data indicated that CFO-20 electrodes exhibited the best pseudocapacitive performance. Impedance spectroscopy data showed high imaginary part of impedance for CFO-0, which was due to low capacitance (Figure 7A). The high real part of the complex impedance indicated high electrical resistance.

The addition of MCNT resulted in a significant decrease in the imaginary part of the impedance and an increase in the slope of the Nyquist plot, which indicated improved capacitive behavior (Figure 7B). Moreover, a significant reduction in the real part of the impedance showed reduced electrode resistance. The CFO-20 electrodes showed lower resistance compared to other electrodes.

The CFO-0, CFO-10, CFO-20 and CFO-30 electrodes showed capacitances (C_S_′) of 0.01, 1.11, 1.04, 0.94 F cm^−2^, respectively, at a frequency of 10 mHz (Figure 7C). The CFO-20 electrode showed the highest C_S_′ at frequencies above 30 mHz. The CFO-10, CFO-20 and CFO-30 electrodes showed a relaxation-type frequency dispersion of C_S_′. The relaxation frequencies for CFO-10, CFO-20 and CFO-30 electrodes, corresponding to C_S_″ maxima, were found to be 0.05, 0.11 and 0.094 Hz, respectively (Figure 7D). The highest C_S_′ at frequencies above 30 mHz and the highest relaxation frequency of the CFO-20 electrode indicated its improved performance at high charge–discharge rates in agreement with CV data at different scan rates for the same electrode. It should be noted that capacitance obtained from the CV data in a wide potential range (−0.8–0 V) was influenced by a scan rate, whereas the real part of capacitance derived from the impedance data using a low amplitude AC voltage (5 mV) depended on frequency.

**Figure 8:**
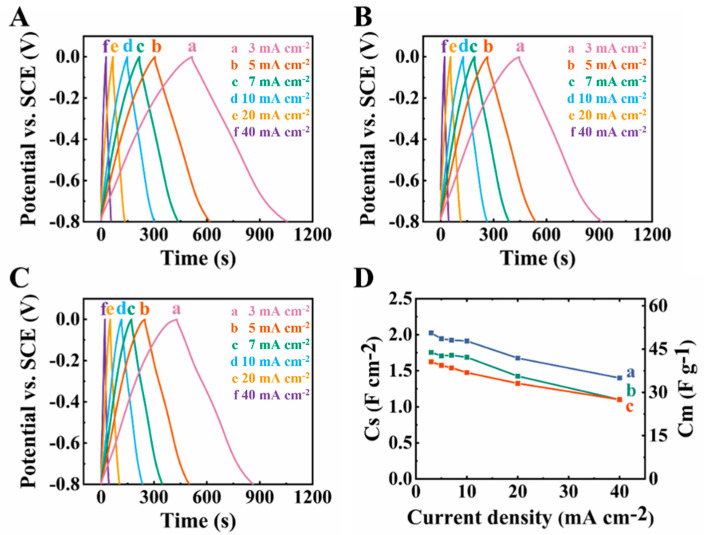
(**A**–**C**) galvanostatic charge discharge data at current densities of (a) 3, (b) 5, (c) 7, (d) 10, (e) 20 and (f) 40 mA cm^−2^ for (**A**) CFO-10, (**B**) CFO-20 and (**C**) CFO-30 electrodes; (**D**) capacitance versus current density for (a) CFO-10, (b) CFO-20 and (c) CFO-30 electrodes.

The capacitive behavior of the electrodes was also analyzed using galvanostatic charge–discharge data at different current densities (Figure 8). The increase in the MCNT content resulted in the improved shape of the charge–discharge curves, which were nearly triangular (Figure 8A–C) for CFO-20 and CFO-30. The capacitances, obtained at 3 mA cm^−2^ were 2.03, 1.76 and 1.62 F cm^−2^ for CFO-10, CFO-20 and CFO-30 electrodes, respectively.

**Figure 9:**
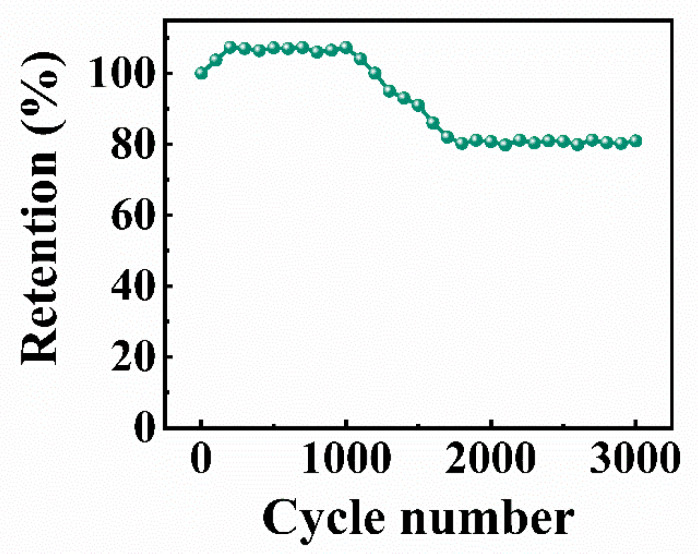
Capacitance retention for CFO-20 electrodes.

This Figure 9 shows cyclic stability data for the CFO-20 electrodes. The increase in capacitance during the first 200 cycles can be attributed to morphology changes during cycling. A similar increase in capacitance during initial cycling due to internal electrode microstructure changes was observed in other materials [46,47,48,49]. The capacitance decreased after about 1000 cycles, the capacitance retention after 3000 cycles was 81%. The capacitance decrease can be attributed to the partial corrosion of the surface of the electrode material.

Table 1 compares the experimental results of this work with literature data of other investigations. Capacitive properties of CuFe_2_O_4_ were mainly investigated in a positive potential range [50,51,52,53,54] or relatively narrow negative and positive potential ranges [55]. The charging mechanism in the positive potential range [51] involved the decomposition of CuFe_2_O_4_ to form individual oxides CuO and Fe_2_O_3_. It is not clear if a reverse reaction at room temperature can result in the synthesis of CuFe_2_O_4_. CV data showed well-defined redox peaks indicating battery-type behavior [50,51,52,53,54,56] in a relatively narrow potential window. The galvanostatic charge–discharge curves deviated significantly [50,51,52,53] from the ideal triangular shape of the capacitor materials. CuFe_2_O_4_ electrodes were tested in KOH [50,51,52,54,55] and H_2_SO_4_ [53] electrolytes. CuFe_2_O_4_-based electrodes showed high resistance, which is detrimental for the development of electrodes and devices with high power density [50,52,53].

In contrast to previous investigations, which reported battery-type behavior in the positive potential range, in our investigation we observed pseudocapacitive behavior in the negative potential range. Good capacitive behavior was achieved at a high active mass of 40 mg cm^−2^. Recent studies [5,57] have highlighted the need for the development of efficient electrodes with active mass loading above 10–20 mg cm^−2^ for practical applications. Investigations have also revealed [5] the significant influence of active material mass loading on mass normalized capacitance, which reduced by 2–3 orders of magnitude with increasing active mass from several μg cm^−2^ to the level of 10–20 mg cm^−2^. High active mass loading is important for reducing the contribution of inactive components to the total mass of the electrodes and devices. However, it is challenging to achieve good material performance at high active mass. The approach developed in this investigation allowed for high areal capacitance in a Na_2_SO_4_ electrolyte. In this investigation, MCNTs were used as conductive additives. It is known that MCNTs exhibit low capacitance [58] due to their low surface area, compared with activated carbons, graphene and other advanced carbon materials. Due to the low gravimetric capacitance of pure MCNTs, the reduction in capacitance with the increasing active mass of the composite material, and the low MCNT content of the composites (e.g., 10% in CFO-10), the contribution of the double layer capacitance of MCNTs to total capacitance was negligibly small and the pseudocapacitive properties of the composites were mainly attributed to the pseudocapacitive properties of the CuFe_2_O_4_ material.

Despite the use of electrodes with high active mass, the electrode resistance was significantly lower than the resistance of the CuFe_2_O_4_ electrodes reported in previous investigations [50,52,53]. The ability to achieve high capacitance at a low resistance in a relatively large voltage window is promising for the development of devices with enhanced power–energy characteristics. Comparison with the literature data [5] indicated that the areal capacitance of the CuFe_2_O_4_-based electrodes is on par with the best negative electrodes for operation in an environmentally friendly Na_2_SO_4_ electrolyte. Areal capacitance is an important parameter for matching negative and positive electrodes and the optimization of device performance. The pseudocapacitive properties of CuFe_2_O_4_ observed in this investigation coupled with the advanced magnetic properties of this material make it a promising MOPC material. It is suggested that the pseudocapacitive properties of CuFe_2_O_4_ in the negative potential range are related to the reduction in Cu^2+^ and Fe^3+^ ions. The reduction process can result in changes in material magnetization. Therefore, the results of this work provide a platform for the investigation of phenomena related to the relationship between magnetic and pseudocapacitive properties of MOPC materials. It should be noted that the electrodes developed in this investigation cannot be used for the observation of magnetocapacitive phenomena. One of the key factors for the observation of pseudocapacitive properties of CuFe_2_O_4_ is related to the use of advanced - Ni foam current collectors which exhibit high corrosion resistance, high conductivity and high porosity, and facilitate the fabrication of electrodes with high active mass and low contact resistance. However, Ni is a ferromagnetic material with relatively high magnetization. It is magnetized under the influence of an external magnetic field and creates its own magnetic field. Therefore, the use of magnetic current collectors must be avoided. An important challenge is the development of special non-magnetic current collectors with high conductivity, high corrosion resistance and high porosity, which will allow for the good utilization of pseudocapacitive properties of CuFe_2_O_4_. Other challenges are related to Lorentz forces, which can influence the ion diffusion and current response.

## 3. Materials and Methods

CuFe_2_O_4_ nanopowder (particle size <100 nm), ASPA, Na_2_SO_4_ and polyvinyl butyral-co-vinyl alcohol-co-vinyl acetate (PVBAA, Mw = 65 kDa) were purchased from Millipore Sigma (Oakville, ON, Canada). Multiwalled carbon nanotubes (MCNT, diameter 13 nm, length 1–2 μm) were supplied by Bayer Corp (Leverkusen, Germany.

Suspensions containing CuFe_2_O_4_, conductive MCNT additives and ASPA as a co-dispersing agent were prepared under probe sonication for 5 min. The mass of ASPA was 15% of the total mass of CuFe_2_O_4_ and MCNT. The content of individual components, such as commercial MCNT and commercial CuFe_2_O_4_, in the composites was varied by their mixing in a desired ratio. The masses of MCNT in the CuFe_2_O_4_-MCNT composites CFO-0, CFO-10, CFO-20 and CFO-30 were 0, 10, 20 and 30 wt.%, respectively. The electrodes were fabricated by impregnating Ni foam (95% porosity, Vale, Mississauga, ON, Canada) current collectors with ethanol slurries containing CuFe_2_O_4_, MCNT and PVBAA as the binder. The binder content was 3% of the total mass of CuFe_2_O_4_ and MCNT. The thickness, mass loading and area of the electrodes were 0.38 mm, 40 mg cm^−2^ and 1 × 1 cm^2^, respectively. The structures and morphologies of the composites were characterized by scanning electron microscopy (SEM, JEOL JSM-7000F, Austin, TX, USA), transmission electron microscopy (TEM, Talos 200X, Thermo Fisher Scientific, Waltham, MA, USA) and X-ray diffraction analysis (Bruker Smart 6000 X-ray diffractometer, Bruker, Billerica, MA, USA, CuKα radiation). The magnetic measurements were performed using a Quantum Design Magnetic Properties Measurement System (MPMS, San Diego, CA, USA,). Electrochemical impedance spectroscopy (EIS) and cyclic voltammetry (CV) investigations were conducted using a potentiostat (AMETEK 2273, Berwyn, US). Galvanostatic charge discharge (GCD) was performed by Biologic AMP 300 (Biologic, Willow Hill, IL, USA). The electrochemical analysis was performed in a three-electrode setting, with a large surface area Pt gauze and a saturated calomel electrode (SCE) as the counter and reference electrodes, respectively. The electrodes were analyzed in a potential range between −0.8 and 0 V in 0.5 M Na_2_SO_4_ aqueous electrolyte. The areal (Cs)- and gravimetric (Cm)-specific capacitances were calculated from CV, EIS and GCD data, as described in previous investigations [5,59,60].

The capacitance was calculated from the cyclic voltammetry (CV) data:

(1) $$C=\frac{\Delta Q}{\Delta U}=\frac{\left|\int_{0}^{t\left(Umax\right)}Idt|+|\int_{t\left(Umax\right)}^{0}Idt\right|}{2Umax}$$

where Δ*Q* denotes charge, *I* denotes current and Δ*U* denotes the potential range, and from the chronopotentiometry data:

C = *I*Δ*t*/Δ*U* (2)

The differential complex capacitance C*(ω) = C′(ω) − *i*C″(ω) was calculated at different frequencies (ω) from the complex impedance Z*(ω) = Z′(ω) + *i* Z″(ω) data:

(3) $$C^{\prime }\left(\omega \right)=\frac{-Z^{\prime \prime }\left(\omega \right)}{\omega |Z{\left(\omega )\right|}^{2}}$$

(4) $$C^{\prime \prime }\left(\omega \right)=\frac{Z^{\prime }\left(\omega \right)}{\omega |Z{\left(\omega )\right|}^{2}}$$

Following the goal of this investigation, we analyzed capacitive properties by cyclic voltammetry, impedance spectroscopy and galvanostatic charge–discharge methods and demonstrated the good cyclic stability of the CuFe_2_O_4_ electrodes.

## 4. Conclusions

This investigation revealed the pseudocapacitive properties of CuFe_2_O_4_. A high areal capacitance of 2.76 F cm^−2^ was achieved at a low resistance in a relatively large negative potential window, which makes CuFe_2_O_4_ a promising negative electrode for the development of supercapacitors operating in an environmentally friendly Na_2_SO_4_ electrolyte. The approach developed in this investigation allowed good material performance at high active mass loading, which is important for practical applications. It was based on the use of ASPA as a chelating co-dispersant for CuFe_2_O_4_ and MCNT. The ASPA adsorption on CuFe_2_O_4_ and MCNT involved different mechanisms which were linked to features of the ASPA structure. CuFe_2_O_4_ nanoparticles combined magnetic ordering and advanced pseudocapacitive properties, making CuFe_2_O_4_ a promising MOPC material. The combination of advanced magnetic and capacitive properties of CuFe_2_O_4_ in the negative potential range provides a platform for the investigation of new phenomena related to the influence of pseudocapacitive/magnetic properties on magnetic/pseudocapacitive behavior.

## Figures and Tables

**Table 1:** Properties of CuFe_2_O_4_ electrodes.

Mass Loading	Capacitance or Capacity	Scan Rate or Current Density	Potential Range	Electrolyte	Cyclic Stability	Cycle Number	Reference
^§^	*1940 Fg^−1^ 1164 Cg^−1^	1 Ag^−1^	0–0.6 V vs. Ag/AgCl	6 M KOH	98%	10,000	[50]
^§^	*334 Fg^−1^	0.6 Ag^−1^	−0.1–+0.5 V vs. SCE	1 M KOH	88%	600	[51]
^§^	*189.2 Fg^−1^	0.5 Ag^−1^	−0.1–+0.6 V vs. Ag/AgCl	2 M KOH	84%	1000	[52]
3 mg cm^−2^	*437.3 Fg^−1^	0.004 Vs^−1^	0.15–0.75 V vs. SCE	0.5 M H_2_SO_4_	88.6%	2000	[53]
0.3 mg cm^−2^	250.8 Fg^−1^	2 mVs^−1^	–0.25–0.35 V vs. Ag/AgCl	3 M KOH	>90%	1000	[55]
40 mg cm^−2^	2.76 F cm^−2^ (69 Fg^−1^)	2 mVs^−1^	−0.8–0 V vs. SCE	0.5 M Na_2_SO_4_	81%	3000	This work

^§^ not presented in the reference; * battery-type behavior.

## Data Availability

The data is provided in this paper and Supplementary Material.

## Supplementary Materials

The following supporting information can be downloaded at: https://www.mdpi.com/article/10.3390/molecules27165313/s1, Figure S1: SEM image of CFO-20 electrode and EDS mapping results for the selected area in the SEM image.

## Sample Availability

Samples of the compounds are available from the authors.
