# Supplementary material for: Magnetic CuFe2O4 Nanoparticles with Pseudocapacitive Properties for Electrical Energy Storage
Source: Molecules. 2022 Aug 20;27(16):5313. doi: 10.3390/molecules27165313 (PMC9413230; doi:10.3390/molecules27165313)
Supplement: Supplementary file 1 [file molecules-27-05313-s001.zip › molecules-1829413-supplementary.pdf]

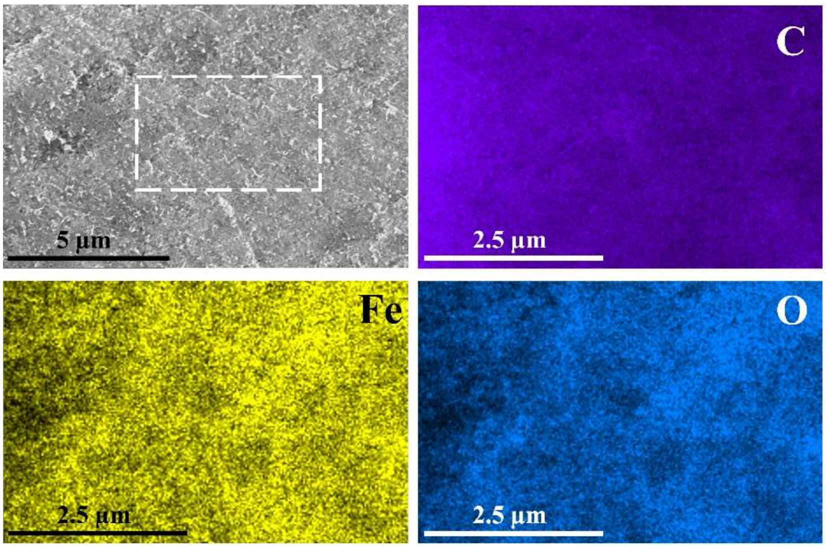

Figure S1. SEM image of CFO-20 electrode and EDS mapping results for the selected area in the SEM image.
